# Expedited Partner Therapy (EPT) increases the frequency of partner notification among MSM in Lima, Peru: a pilot randomized controlled trial

**DOI:** 10.1186/s12916-017-0858-9

**Published:** 2017-05-04

**Authors:** Jesse L. Clark, Eddy R. Segura, Catherine E. Oldenburg, Jessica Rios, Silvia M. Montano, Amaya Perez-Brumer, Manuel Villaran, Jorge Sanchez, Thomas J. Coates, Javier R. Lama

**Affiliations:** 1 David Geffen School of Medicine, University of California, Los Angeles, CA USA; 2 Escuela de Medicina, Universidad de Ciencias Aplicadas, Lima, Peru; 3 Francis I. Proctor Foundation, University of California, San Francisco, CA USA; 4 Asociacion Civil Impacta Salud y Educacion, Lima, Peru; 5 US Naval Medical Research Unit-6, Callao, Peru; 6 Columbia University Mailman School of Public Health, New York, NY USA; 7 Universidad Nacional Mayor San Marcos, Centro de Investigaciones Tecnológicas, Biomédicas y Medioambientales, Lima, Peru; 8 Department of Medicine, Division of Infectious Diseases, UCLA Geffen School of Medicine, 10833 Leconte Avenue, CHS 37-121, Los Angeles, CA 90095 USA

**Keywords:** Expedited Partner Therapy, Partner notification, MSM, Latin America

## Abstract

**Background:**

Expedited Partner Therapy (EPT) has been shown to improve treatment outcomes among heterosexual partners of individuals with curable sexually transmitted infections (STIs). Although the use of EPT with men who have sex with men (MSM) has been debated, due to the potential for missed opportunities to diagnose unidentified cases of HIV and syphilis infection in symptomatic partners, increases in partner notification (PN) resulting from use of EPT may promote testing and treatment of otherwise unidentified partners. We assessed the impact of EPT on self-reported PN among MSM in Peru with gonorrheal (GC) and/or chlamydial (CT) infection.

**Methods:**

We enrolled 173 MSM in Lima, Peru with symptomatic or asymptomatic GC and/or CT infection between 2012 and 2014. We enrolled 44 MSM with symptomatic urethritis/proctitis and 129 MSM with asymptomatic GC/CT infection, diagnosed based on nucleic acid testing (Aptima Combo 2 Transcription-Mediated Amplification [TMA]) from urethral, pharyngeal, and rectal sites. Eligible participants were randomly assigned to receive either standard PN counseling (*n* = 84) or counseling plus EPT (cefixime 400 mg/azithromycin 1 g) for up to five recent partners (*n* = 89). Self-reported notification was assessed by computer-assisted self-administered survey among 155 participants who returned for 14-day follow-up.

**Results:**

The median age of participants was 26 (interquartile range [IQR]: 23–31) with a median of 3 sexual partners (IQR: 2–4) in the previous 30-day period. Among all participants, 111/155 (71.6%) notified at least one partner at 14-day follow-up with a median of 1 partner notified per participant (IQR: 0–2). For participants randomized to receive EPT, 69/83 (83.1%) reported notifying at least one partner, compared with 42/72 (58.3%) of participants in the control arm (odds ratio = 3.52; 95% confidence interval [CI]: 1.68–7.39). The proportion of all recent partners notified was significantly greater in the EPT than in the control arm (53.5%, 95% CI: 45.0–62.0% versus 36.4%, 95% CI: 27.0–47.4%).

**Conclusions:**

Provision of EPT led to significant increases in notification among Peruvian MSM diagnosed with GC/CT infection. Additional research is needed to assess the impact of EPT on biological outcomes, including persistent or recurrent infection, antimicrobial resistance, and HIV/STI transmission, in MSM sexual networks.

**Trial registration:**

ClinicalTrials.gov, NCT01720654. Registered on 10/29/2012.

## Background

Expedited Partner Therapy (EPT) provides an opportunity for the targeted delivery of sexually transmitted infection (STI) control interventions to high-risk sexual partnerships and networks. By providing antibiotic therapy to the recent partners of STI-positive index patients, either through patient delivery or alternate methods of expedited access without a prescription, EPT removes key institutional and interpersonal barriers to treatment [1–3]. By providing direct access to antibiotics for an index patient’s recent sexual partners, EPT alleviates structural barriers like limited access to clinical services [4, 5]. At the same time, by redirecting the public act of formally seeking STI care into a private interaction between sexual partners, EPT also circumvents social barriers to partner testing and treatment like stigma and shame [6–8]. Previous clinical trials of EPT for partner management of urethral gonorrhea (GC), chlamydia (CT), and other bacterial STIs found that individuals randomized to receive EPT had significant reductions in the frequency of repeat or recurrent infection on subsequent re-testing [9–14]. By providing a tool to support partner notification following an STI diagnosis, EPT has also been shown to promote notification of recent sexual partners [15–18]. As a result, the US Centers for Disease Control (CDC) currently recommends routine use of EPT for heterosexual men and women diagnosed with GC and/or CT infection [19, 20]. However, lingering questions concerning the use of EPT with sexual partners of men who have sex with men (MSM) have discouraged regular use of EPT in this population.

Most concerns surrounding the provision of EPT for sexual partners of MSM are based on the high prevalence of undiagnosed HIV, syphilis, and drug-resistant gonorrhea infections within their sexual networks [21, 22]. Public health concerns that providing direct, partner-delivered access to oral antibiotic therapy for STI-exposed individuals may result in ineffective treatment and discourage them from seeking further testing and treatment services have impeded the introduction of EPT as a strategy for partner management of MSM with GC/CT infection [23]. The potential for the increased development of population-level antibiotic resistance further complicates empiric antibiotic use for exposed partners [24, 25]. Clinical evidence on the use of EPT with MSM is limited; the only previous randomized controlled trial (RCT) of EPT with partners of MSM was discontinued prior to completion due to a low rate of subject enrollment [26, 27]. Accordingly, while EPT is recommended for use with MSM by the California Department of Public Health, national guidelines are inconsistent, and the CDC currently recommends caution in the use of EPT for partners of MSM, citing the need for additional clinical trial research prior to widespread use in this population [19, 28].

Another key issue affecting EPT with MSM are the variations in male same-sex partnership interactions between different social and cultural contexts. The majority of research on EPT has been conducted with heterosexual partnerships in the USA and Western Europe, with little attention paid to how the delivery of patient-delivered partner therapy might differ within the interpersonal dynamics of male and/or transgender female sexual partnerships in developing country settings [29–32]. Social, cultural, and structural differences in how same-sex sexual partnerships are defined, how norms of gender and sexuality influence power dynamics and communication patterns within these partnerships, and how access to healthcare services affects HIV/STI testing and treatment outcomes are all critical issues to address prior to the large-scale introduction and global dissemination of EPT and other partner-based HIV and STI prevention interventions [33–38].

As a result, it is critical to understand how EPT impacts HIV and STI transmission within male same-sex sexual partnerships, not only in terms of individual-level outcomes like persistence or recurrence of bacterial infections, but also in terms of partner- and network-level outcomes like frequency of partner notification, testing, and treatment for HIV and other STIs among both directly and indirectly linked members of at-risk networks. Previous systematic reviews and meta-analyses have suggested that EPT (as well as other practical tools to support partner notification efforts) increases the likelihood of notification, thereby also increasing the probability that sexual partners will be encouraged to seek formal HIV/STI counseling and testing [3, 4, 39, 40]. However, none of these outcomes has been fully evaluated in RCTs of MSM sexual partnerships or in developing country settings.

Lima, Peru provides a valuable scientific environment in which to explore questions of STI control and transmission within sexual networks of MSM. The HIV epidemic in Peru is concentrated among MSM and transgender women (TW), with relatively few cases of HIV ascribed to heterosexual contact or injection drug use [41, 42]. At the same time, new HIV diagnoses are frequently accompanied by STI co-infection, suggesting that behavioral and/or biological factors support HIV/STI co-transmission in this population [43, 44]. Finally, social constructions of sexual identity influence interpersonal dynamics, sexual practices, and risks for HIV and STI transmission within MSM partnerships and networks [45]. In order to assess the effect of partner-delivered antibiotic therapy on partner notification outcomes among MSM in Lima, Peru, we conducted a pilot RCT of EPT among MSM diagnosed with rectal, pharyngeal, or urethral GC and/or CT infection.

## Methods

Between October 2012 and July 2014, we conducted an RCT to assess the effect of Expedited Partner Therapy (EPT) on self-reported partner notification outcomes among MSM in Lima, Peru diagnosed with symptomatic urethritis and/or proctitis or asymptomatic gonococcal and/or chlamydial infection at any anatomic site (oral, rectal, or pharyngeal).

### Screening procedures

Potential participants were recruited for STI screening from community and clinic sites by staff recruiters of Asociacion Civil Impacta Salud y Educacion, a non-profit HIV and STI research center in Lima, Peru. Men and transgender women (TW) who reported anal intercourse with at least one male or transgender female partner in the previous 6 months were eligible for the screening protocol. Participants in the screening study completed a computer-assisted self-administered (CASI) survey that addressed demographic characteristics, history of HIV and STIs, use of alcohol and drugs attitudes, sexual network characteristics, and attitudes, beliefs, and community norms surrounding partner notification for HIV and STIs. The survey also asked for detailed characteristics of participants’ three most recent sexual partners, including each partner’s gender and sexual identity, partner-specific sexual practices, and the likelihood of notifying each partner following an STI diagnosis. In order to assist with future recall of partner data, participants were asked to identify each of these partners with a Nickname or Other Identifying Characteristic (e.g., “the guy in the blue shirt from *La Cueva*”).

Screening study participants underwent a physical examination to identify signs of symptomatic urethritis or proctitis (urethral or rectal discharge and/or inflammation) as well as primary or secondary syphilis infection (ulcerative lesions on oral, anal, or genital mucosa or macular rash characteristic of secondary syphilis). Following clinical evaluation, rectal and pharyngeal swabs as well as urine samples were collected to test for urethral, rectal, and pharyngeal gonorrhea and/or chlamydia infection by nucleic acid testing (Aptima Combo 2 Transcription-Mediated Amplification [TMA], Hologic, Marlborough, MA, USA). Blood was collected to test for syphilis infection by rapid plasma reagin (RPR) assay (RPRnosticon, Biomérieux, Marcy l’Etoile, France) with microhemagglutination assay for *Treponema pallidum* (MHA-TP) confirmation (MHA-TP, Organon Teknika, Durham, NC, USA) and serial dilution of RPR titers for positive results. All participants were offered free HIV testing, though it was not required as a condition of enrollment. All TMA samples were tested at the US Naval Medical Research Unit-6 Bacteriology Laboratory in Callao, Peru, and HIV and syphilis testing were conducted at the Impacta laboratory. Results of all assays were provided within 2 weeks.

All participants with symptomatic urethritis, proctitis, or laboratory-diagnosed GC infection were treated with single doses of ceftriaxone (250 mg delivered intramuscularly [IM]) and azithromycin (1 g by mouth [PO]), while participants with only asymptomatic CT infection were treated with azithromycin (1 g PO). Participants with syphilis infection were treated according to the stage of infection, as determined by the study physician according to their history of syphilis infection, prior RPR titer(s), and antibiotic treatment history. Participants with newly diagnosed HIV infection were referred to local HIV treatment centers designated by the Peruvian Ministry of Health.

### Randomization and enrollment

MSM and TW who reported anal intercourse with at least one male or TW partner in the previous 6 months and who were diagnosed with symptomatic urethritis and/or proctitis, or with asymptomatic urethral, rectal, and/or pharyngeal GC/CT infection were invited to enroll in the EPT trial. Participants with symptomatic infection were enrolled at their initial screening visit, while participants with laboratory-diagnosed infection were enrolled after receiving results of their nucleic acid testing and appropriate antibiotic therapy at the 2-week follow-up screening visit. Participants diagnosed by laboratory assay with both asymptomatic syphilis and GC/CT infection were assigned on an alternating (one-to-one) basis to either the EPT study or to a parallel trial assessing new technologies for partner notification for MSM with syphilis infection. Due to similarities in assessments and outcomes, no co-enrollment between the two trials was permitted. All participants provided signed informed consent to participate in a study on “If and how men with an STI inform their recent sexual partners of their diagnosis.” After providing signed consent, participants were allocated to either intervention or control arms using a previously designed 200-unit randomization scheme generated by the site www.random.org. Allocation assignments were concealed in sealed, opaque, sequentially numbered envelopes that were opened in numerical order by the study counselor at the point of randomization. No deviation from the allocation order or wasting of randomization envelopes was reported.

### Intervention and control procedures

Each randomization envelope contained an assignment to either the intervention or the control arm and a standardized script, to be read by the counselor verbatim. The counseling script advised the participant of the importance of notifying their recent partner of their STI diagnosis and informed them of the availability of free testing and treatment resources at the study site, as well as other area health centers. The counseling script also reminded participants that their safety was a primary concern, and that they should not attempt to notify any partners who they feared might respond with abuse or violence. Participants randomized to the intervention arm were then referred to the on-site pharmacy where they were provided with up to five partner treatment packets, depending on the number of recent partners reported. Each packet included single-dose tablets of cefixime (400 mg) and azithromycin (1 g), printed information about symptoms and sequelae of GC/CT infection, the locations and operating hours of local sites offering free or low-cost HIV/STI testing and treatment services, and a warning against taking the medication in case of a pre-existing allergy to cephalosporins or macrolides. Each packet also included a bold-print message advising recipients that they were being provided with the medication because their recent sexual partner had been diagnosed with GC/CT infection and that they were at high risk for HIV and other STIs. The message advised the recipient to seek professional testing for these infections and *only* to use the medication included if they were unwilling or unable to seek testing and treatment from a local healthcare facility.

### Follow-up procedures

Participants in the intervention and control arms were asked to return to the clinic in 14–21 days for a follow-up evaluation. At the follow-up visit, participants underwent a physical examination to assess for signs of symptomatic infection and repeat GC/CT testing from all three anatomic sites (pharyngeal swab, rectal swab, and first-catch urine). Participants completed a follow-up CASI survey to assess how many of their recent partners (from the 30-day period preceding their diagnosis) had been notified, as well as whether each of their three most recent partners had been notified and received treatment. Participants were reminded of the total number of prior sexual partners they had reported at the baseline visit and to quantify how many of these partners had been notified. To assist with recall of recent partner data, participants were reminded of the Nickname or Other Identifying Characteristic they had assigned each of their three most recent partners, as well as the partner’s gender and sexual identity. Survey questions used a 4-point Likert scale to assess whether each partner had been notified, whether the participant provided them with antibiotic treatment (for participants in the intervention arm only), whether the partner had taken any antibiotic treatment (for all participants), whether the partner had sought HIV/STI testing, and the participant’s level of certainty regarding each outcome.

### Sample size and power calculations

Sample size calculations were based on partner notification outcomes from previous observational studies of Peruvian men and women diagnosed with HIV or STI [33, 46]. Assuming a baseline frequency of 56% for notification of any partner, a sample of 170 subjects was projected to have 80% power to detect a 20% increase in notification of any recent partner.

### Statistical analysis

Descriptive characteristics by study arm were calculated with medians and interquartile ranges (IQR) for continuous variables and proportions for categorical variables. The primary outcome was self-reported notification of any recent sexual partner after 14 days of follow-up. The proportion of participants reporting notification of any partner versus notification of no partners in the entire study sample and the proportion among those reporting at least one partner were calculated by study arm, and a logistic regression model was used to calculate odds ratios (ORs). Secondary outcomes included the proportion of all recent partners notified and participant and partner characteristics associated with partner testing and treatment. We calculated the percentage of all partners, male stable partners, and male casual partners who were notified by dividing the total number of partners reported for each category by the total number of partners notified for each category. We used a Wilcoxon rank-sum test to compare the percentage of partners notified by study arm. Finally, we used a logistic generalized estimating equation model to assess notification and treatment outcomes by study arm for the last three partners for each participant, including: (1) if the partner was notified; (2) if the participant knows the partner received the message; (3) if the partner was observed taking antibiotics; (4) if the partner was tested for HIV and/or STIs; and (5) if the partner received any medical treatment (regardless of the source of treatment). No interim analyses were conducted. All analyses were conducted in Stata 14.1 (StataCorp, College Station, TX, USA).

### Human subjects protections

All study procedures were reviewed and approved by the UCLA Office for Human Research Participant Protection (IRB 11-003095), the *Asociacion Civil Impacta Comite de Bioetica* (Certificate 0053-2012-CE), and the US Naval Medical Research Unit-6 (Protocol HRPP NAMRU6.2012.0033), and registered with the Peruvian *Instituto Nacional de Salud* prior to the initiation of any activities. All participants underwent separate informed consent procedures for the screening and RCT protocols and provided written informed consent for each protocol. The clinical trial was registered with www.ClinicalTrials.gov (Protocol Number NCT01720654).

## Results

**Fig. 1:**
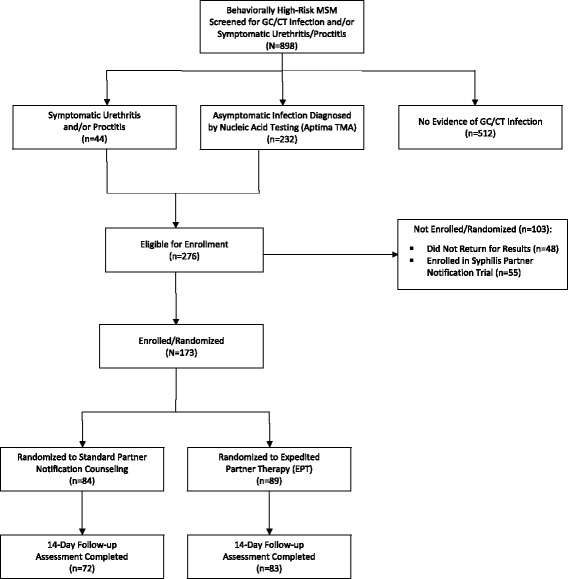
Screening, enrollment, and follow-up Consolidated Standards of Reporting Trials (CONSORT) flowchart; Lima, Peru 2012–2014

We screened a total of 898 individuals, of whom 276 met criteria for enrollment (Fig. 1). Symptomatic urethritis and/or proctitis was noted in 44 participants, and asymptomatic, laboratory-diagnosed infection was present in 232 (13 participants diagnosed with symptomatic urethritis/proctitis subsequently tested negative for GC/CT infection by TMA). Of the 276 eligible individuals, 55 were enrolled in a parallel trial of partner notification for syphilis infection, and 48 did not return for their test results. We enrolled 173 subjects into the intervention (*n* = 89) and control (*n* = 84) arms between October 2012 and June 2014.

Baseline characteristics of participants in the intervention and control arms are presented in Table 1. In the control arm, 72/84 subjects (85.7%; 95% confidence interval [95% CI]: 76.6–91.6%) returned for the 14-day follow-up visit, compared with 83/89 subjects (93.2%; 95% CI: 86.0–96.8%) in the EPT arm. In both groups, the median age was 26, with most participants having completed some college or technical school. The majority in both arms identified as gay or homosexual, with a median of three male sexual partners reported during the previous 30-day period. In both arms, the most common anatomic site of infection was rectal (53.6% [95% CI: 43.0–63.8%] in the control arm; 46.1% [95% CI: 36.1–56.4%] in the intervention arm), though a higher prevalence of urethral infection was observed in the intervention (29.2%; 95% CI: 20.8–39.4%) than the control (14.3%; 95% CI: 8.4–23.3%) group. There was no significant difference in the frequency of symptomatic infection between the control (23.8%; 95% CI: 16.0–33.9%) and the intervention (27.0%; 95% CI: 18.8–37.0%) arms (Table 2).

**Table 1:** Baseline characteristics by randomization arm; Lima, Peru, 2012–2014

	Randomization arm	
	Control (*n* = 84)	Expedited Partner Therapy (EPT) (*n* = 89)
Age (median, IQR)	26 (22–31)	26 (23–32)
Education		
Primary	1 (1.1%)	2 (2.2%)
Incomplete secondary	11 (13.1%)	6 (6.7%)
Complete secondary	17 (20.2%)	23 (25.8%)
University or vocational training	55 (65.5%)	58 (62.9%)
Sexual identity		
Heterosexual	3 (3.6%)	6 (6.7%)
Bisexual	22 (26.2%)	23 (25.8%)
Homosexual/gay	57 (67.8%)	51 (58.4%)
Trans	0 (0%)	1 (1.1%)
Other	2 (2.4%)	3 (3.4%)
I don’t know	0 (0%)	4 (4.5%)
Sexual role		
*Activo* (insertive)	17 (20.2%)	22 (24.7%)
*Pasivo* (receptive)	24 (28.6%)	25 (28.1%)
*Moderno* (versatile)	40 (47.6%)	37 (41.6%)
Other	3 (3.6%)	3 (3.4%)
I don’t know	0 (0%)	2 (2.2%)
Number of sexual partners, 30 days (median, IQR)	3 (3–4)	3 (3–5)
Number of male partners	3 (3–4)	3 (2–5)
Number of female partners	0 (0–0)	0 (0–0)
Symptomatic infection	20 (23.8%)	24 (27.0%)
Returned for follow-up evaluation	72 (85.7%)	83 (93.2%)

**Table 2:** Prevalence and anatomic site of gonorrhea (GC) and chlamydia (CT) infections at baseline and follow-up visits

Anatomic site of infection	Control arm		EPT arm	
	Baseline (*N* = 84)^a^	Follow-up (*N* = 72)	Baseline (*N* = 89)	Follow-up (*N* = 83)
Any site/any pathogen	*n* = 79^b^ 94.0% (86.8–97.4%)	*n* = 2^c^ 2.8% (0.8–9.6%)	*n* = 81 91.0% (83.2–95.4%)	*n* = 2 2.4% (0.7–8.4%)
Urethral CT	*n* = 4 5.1% (2.0–12.3%)		*n* = 10 11.2% (6.2–19.5%)	
Urethral GC	*n* = 6 7.1% (3.3–14.7%)		*n* = 11 12.4% (7.0–20.8%)	
Urethral CT and GC	*n* = 2 2.4% (0.6–8.3%)		*n* = 5 6.2% (2.7–13.6%)	
Rectal CT	*n* = 24 28.6% (20.0–39.0%)	*n* = 2 2.8% (0.8–9.6%)	*n* = 32 36.0% (26.8–46.3%)	*n* = 1 1.2% (0.2–6.5%)
Rectal GC	*n* = 12 14.3% (8.4–23.3%)		*n* = 4 4.5% (1.8–11.0%)	
Rectal CT and GC	*n* = 9 10.7% (5.7–19.1%)		*n* = 5 6.2% (2.7–13.6%)	
Pharyngeal CT	*n* = 8 9.5% (4.9–17.7%)		*n* = 1 7.9% (3.9–15.4%)	
Pharyngeal GC	*n* = 15 17.9% (11.1–27.4%)		*n* = 21 25.9% (17.6–36.4%)	*n* = 1 1.2% (0.2–6.5%)
Pharyngeal CT and GC	*n* = 1 1.2% (0.2–7.4%)		*n* = 1 1.2% (0.2–6.7%)	

^a^Subtotals do not add to 100% as individual participants may have had multiple pathogens and/or anatomic sites of infection

^b^Five subjects enrolled in the control arm and eight participants in the EPT arm diagnosed with symptomatic disease subsequently tested negative for GC/CT infection by TMA

^c^Only cases of infection with the same organism in the same anatomic site at follow-up reported

Among all participants completing follow-up, 111/155 (71.6%) notified at least one partner, with a median of 1 partner notified per participant (IQR: 0–2). The proportion of participants who reported notifying any of their recent sexual partners at the 14-day follow-up was 83.1% (95% CI: 73.6–90.1%) in the EPT arm and 58.3% (46.8–69.2%) in the control arm (OR = 3.52, 95% CI: 1.68–7.39) (Table 3).

**Table 3:** Partner notification outcomes among MSM with gonorrhea and/or chlamydia infection

	Expedited Partner Therapy (EPT) (*n* = 83)	Standard partner notification counseling (*n* = 72)	Odds ratio (95% CI)
Proportion of participants who notified any recent partners	83.1% (69/83)	58.3% (42/72)	3.52 (1.68, 7.39)
Proportion of participants who notified any recent partners (only participants reporting ≥1 recent partner)	85.2% (69/81)	61.8% (42/68)	3.56 (1.62, 7.80)

When subjects who reported no sexual partners during the previous 30 days were excluded from the analysis, the proportion of participants who had notified any recent partner increased to 85.2% (75.8–91.3%) and 61.8% (50.0–72.4%) in the intervention and control groups, respectively (OR = 3.56, 95% CI: 1.62–7.80). The proportion of all recent partners who had been notified was also higher among participants assigned to receive EPT (53.5%; 95% CI: 45.0–62.0%) than among participants who received only standard counseling (36.4%; 95% CI: 27.0–45.9%; *p* = 0.004). No episodes of abuse, violence, or other adverse reactions to notification were reported.

**Fig. 2:**
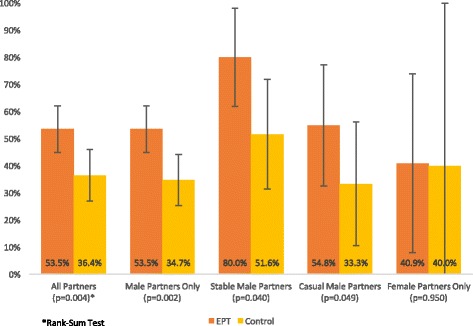
Proportion of all recent partners notified among MSM diagnosed with GC/CT infection; Lima, Peru 2012–2014

Notification outcomes varied according to partnership status and partner gender (Fig. 2). While no difference was observed in notification outcomes for female partners, participants who received EPT reported notifying a significantly larger proportion of their male partners (53.5% [95% CI: 45.0–62.0%] versus 34.7% [95% CI: 27.0–47.4%]; *p* = 0.002). As in other studies with similar populations, stable partners were more likely to be notified than casual partners, though EPT was associated with a significantly higher likelihood of notification for both stable and casual partners. In the intervention group, 80.9% (95% CI: 61.9–98.1%) of stable male partners had been notified, compared with 51.6% (95% CI: 31.4–71.8%) in the control group (*p* = 0.04). Similarly, while 54.8% (95% CI: 32.4–77.1%) of casual partners were notified by participants randomized to receive EPT, only 33.3% (95% CI: 10.5–56.2%) of casual partners were notified by participants who received standard notification counseling (*p* = 0.049).

**Fig. 3:**
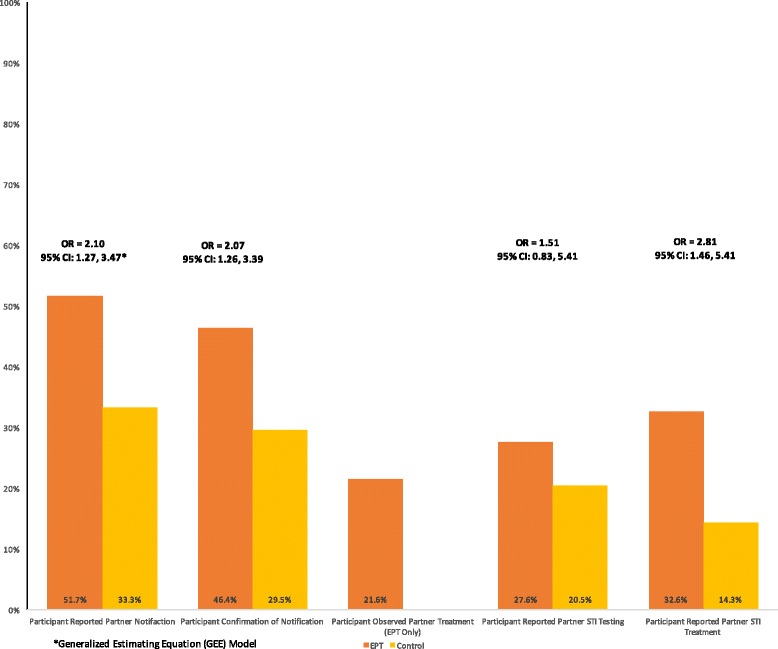
Prevention cascade outcomes of partners of MSM diagnosed with GC/CT infection; Lima, Peru 2012–2014

Analysis of data from the three most recent sexual contacts demonstrates a similar superiority of EPT over standard partner notification (PN) counseling in terms of partner notification, testing, and treatment outcomes (Fig. 3). Participants in the EPT arm were significantly more likely to report notifying at least one of these partners (OR = 2.10; 95% CI: 1.27–3.47), to be certain that the notification message was received by (or delivered directly to) the partner (OR = 2.07; 95% CI: 1.26–3.39), and to know that the partner received some form of medical treatment (either participant-delivered or from a healthcare provider) (OR = 2.81; 95% CI: 1.46–5.41). Participants in the EPT arm also more frequently reported that their partners had been tested for HIV and/or STIs, though this difference was not statistically significant (OR = 1.51; 95% CI: 0.83–2.75). Despite the improvements in the likelihood of partner notification and treatment, we note that even in the EPT arm, the frequency of recent partner testing (27.6%) and treatment (32.6%) remained disappointingly low, and the percentage of partners observed taking partner-delivered antibiotic therapy relatively small (21.6%).

Biological outcomes were similar between randomization arms with 5/83 (6.0%; 95% CI: 2.6–13.3%) participants in the EPT arm and 4/72 (5.6%; 95% CI: 2.2–13.4%) in the control arm diagnosed with persistent or recurrent GC/CT infection at the 14- to 21-day follow-up evaluation (*p* = 0.6). Of note, only two participants in each arm were diagnosed with the same bacterial organism in the same anatomic location. Molecular genotyping, to assess for reinfection with the same bacterial strain, and antimicrobial resistance testing were not performed.

## Discussion

In our RCT of Peruvian MSM diagnosed with gonorrhea and/or chlamydia infection, EPT was associated with a significantly higher likelihood of self-reported partner notification and treatment. Men newly diagnosed with GC/CT infection who were provided with partner antibiotic treatment and information packets were more likely to report notifying at least one of their recent male or TW partners, and to notify a larger proportion of their recent sexual partners, than subjects who received only partner notification counseling. Subjects randomized to receive EPT were also more likely to report that they knew their recent partners had received the notification message, and that their partners had been tested and treated for HIV/STIs. Though there was no significant difference in the prevalence of recurrent or persistent GC/CT infection between the intervention and control groups at the 3-week follow-up, these findings provide support for and direction to efforts to use EPT as a strategy for STI control within the sexual networks of MSM in developing country settings.

The frequency of self-reported partner notification observed in our study should be understood within the context of previously reported data on partner notification in Lima, Peru. In a prior observational study of notification outcomes among high-risk men and women in Peru, the frequency of self-reported notification at 1-year follow-up was 65% for main partners and 10% for casual partners [33]. In a subsequent study of anticipated notification among Peruvian MSM/TW newly diagnosed with HIV and/or STI, subjects reported an intention to notify 52.5% of all recent partners, though actual notification outcomes were not measured [46]. In contrast, our analysis was limited to MSM diagnosed with GC/CT infection and found an overall notification frequency of 34.7% for male and TW partners in the control arm (51.6% for main partners and 33.3% for casual partners). In this context, provision of EPT increased the frequency of self-reported notification to 53.5% overall (80.0% of main partners and 54.8% of casual partners). In addition, the fraction of participants who reported notifying any of their recent partners increased from 61.8% in the control group to 85.2% in the intervention arm. This consistency in the observed effect of EPT on different notification metrics (whether analyzed according to the number of subjects who notified at least one partner, the fraction of recent partners notified, or the likelihood of notification for different partnership types) suggests that the impact of EPT extends across a wide range of participant characteristics and partnership contexts.

Examining partner-specific outcomes for participants’ three most recent sexual contacts allows for analysis of our findings within a cascade of care framework [47, 48]. The HIV cascade of care describes a continuum of testing and treatment outcomes from the initial diagnosis of infection, to linkage and retention in care, through initiation and maintenance of antiretroviral therapy, and ultimately long-term virologic suppression. The cascade of care concept has also been applied to management of HIV pre-exposure prophylaxis, syphilis treatment, and EPT [2, 49–51]. In the EPT cascade, an initial diagnosis of GC/CT infection is followed by provision of EPT, acceptance of partner therapy by the index patient, delivery of treatment to the partner, and finally partner treatment. Applying the EPT cascade to our study, participants provided with partner treatment packets were more likely to notify one or more of their recent partners of their diagnosis, to express confidence that these partners had actually received their notification message, to state that these partners had received some form of medical treatment (partner-delivered or otherwise), and to report that these partners had been tested for HIV and STIs. But the proportion of recent partners who were observed taking partner-delivered antibiotic therapy was relatively low, and there was no observed association of EPT with recurrent or persistent biological infection. These clinical outcomes are likely to have been influenced by the verbal and written instructions included with partner treatment packets that advised against taking the antibiotic treatment provided unless there were no other testing and treatment options available and by the low overall prevalence of recurrent or persistent infection observed and the associated limitations in statistical power to detect significant differences. However, if one strictly evaluates the impact on STI control by the biological endpoint of index patient reinfection, then the effect of EPT in this population of at-risk MSM was negligible.

Our findings suggest that use of EPT in MSM sexual networks may require a reconceptualization of the cascade of care framework, from an emphasis on individual-level partner treatment for prevention of recurrent infection to a focus on network-level issues of partner notification, treatment, and testing to effect community-scale reductions in HIV and STI incidence. In the heterosexual patient populations enrolled in previous EPT trials, cases of persistent or recurrent GC/CT infection typically occurred among subjects whose sexual networks were relatively small and whose main risk for re-exposure derived from their primary sexual partner(s) [9–12]. In these studies, most participants reported only 1–1.5 sexual partners within the prior 60-day period, the majority of whom were “main” or “steady” partners [52, 53]. In this partnership context, where STI transmission and re-transmission typically occur through a closed-loop, dyadic partnership system, EPT significantly increases the likelihood of STI cure and bacterial eradication within the partnership.

In contrast, participants in our sample reported a median of 3 different sexual partners during the previous 30-day period and maintained a diverse range of stable, casual, and anonymous partnership types with varying patterns of recurrent sexual contact. As a result of the large number of diverse sexual partnerships and open network patterns reported by men in our study, STI-diagnosed MSM are likely to continue interacting with a large network of continuously shifting sexual contacts in which the model of partner-delivered therapy to limit index patient reinfection is unlikely to be effective. Instead of focusing efforts on the short-term endpoint of bacterial STI cure, future research could address the potential impact of improved partner notification interventions on indirect outcomes like HIV and STI testing, treatment, and linkage to prevention mechanisms with directly and indirectly connected members of MSM sexual networks [54]. By addressing the intermediate steps within the EPT cascade, our findings highlight the potential benefits of EPT for MSM sexual networks and suggest future strategies to use partner management strategies to affect community-scale patterns of both HIV and STI transmission.

There are several limitations to consider when interpreting our findings. The fact that our findings are based on participant self-report of notification, without independent confirmation by sexual partners, raises the possibility that observed improvements in notification frequency may have been due to social desirability bias in reporting outcomes. However, it is likely that this bias would have affected both arms of the study, resulting in a type II error in favor of the null hypothesis. Measures taken to minimize the risk of bias in reported outcomes included enrollment scripts that informed potential participants only that they were invited to a study of “If and how men with an STI inform their recent sexual partners of their diagnosis” and the use of standardized counseling scripts for both study arms. At the same time, the low frequency of persistent or recurrent GC/CT infection precluded the use of biological measures to determine the effect of EPT on partner notification and treatment outcomes. In addition, our 14- to 21-day follow-up period may have been insufficient to accurately diagnose persistent or recurrent GC/CT infection using highly sensitive nucleic acid testing.

Future research with larger sample sizes should incorporate biological outcomes, possibly including molecular epidemiologic analyses to map transmission networks and monitoring of antimicrobial sensitivity to identify the possibility of undertreatment of resistant organisms. In analyzing outcomes, we analyzed participant data on a per protocol instead of an intention-to-treat (ITT) basis. However, given the higher frequency of loss to follow-up in the control arm, ITT analysis of our data would have resulted in an even greater estimate of the effectiveness of the intervention. Our trial was originally designed as a pilot study, and the relatively small sample was not planned to have sufficient power to detect differences in notification practices aside from the primary self-report outcome. Secondary assessments of participant- and partner-level factors modifying the effect of EPT, including the type of STI diagnosed, presence or absence of symptoms, HIV co-infection, and the gender and sexual identities maintained by participants and their partners, are important but beyond the scope of the data presented. Finally, as only one TW was enrolled in the study, we are not to reach any conclusions regarding this population at the current time. Despite these limitations, our findings suggest that EPT could play an important role in improving HIV and STI prevention outcomes within MSM sexual networks and that future research is needed to apply these preliminary findings to larger samples of both MSM and TW.

## Conclusions

In our trial of EPT for Peruvian MSM newly diagnosed with GC/CT infection, patient-delivered partner therapy was associated with an increase in self-reported partner notification and in HIV/STI prevention outcomes across all partnership types. Additional research is needed to evaluate the effectiveness of EPT in promoting partner notification and testing behavior among MSM and to address important questions regarding how to apply these findings to public health systems for HIV prevention and STI control. Instead of focusing on outcomes of recurrent or persistent bacterial infection within discrete dyadic partnerships, these findings suggest that EPT may best be used with sexual partners of MSM as a strategy to access and introduce new prevention strategies into previously hidden high-risk sexual networks. By reconceptualizing the cascade of care framework to focus on community-level testing and treatment outcomes, our data provides a framework for understanding how EPT can be used as a key component of integrated HIV/STI control strategies within MSM sexual networks.

## References

[1] Shiely F, Hayes K, Thomas KK, Kerani RP, Hughes JP, Whittington WL, Holmes KK, Handsfield HH, Hogben M, Golden MR (2010). Expedited partner therapy: a robust intervention. Sex Transm Dis.

[2] Schillinger JA, Gorwitz R, Rietmeijer C, Golden MR (2016). The expedited partner therapy continuum: a conceptual framework to guide programmatic efforts to increase partner treatment. Sex Transm Dis.

[3] Trelle S, Shang A, Nartey L, Cassell JA, Low N (2007). Improved effectiveness of partner notification for patients with sexually transmitted infections: systematic review. BMJ.

[4] Hogben M, Collins D, Hoots B, O’Connor K (2016). Partner services in sexually transmitted disease prevention programs: a review. Sex Transm Dis.

[5] van Aar F, Schreuder I, van Weert Y, Spijker R, Gotz H, Op de Coul E, Partner Notification Group (2012). Current practices of partner notification among MSM with HIV, gonorrhoea and syphilis in the Netherlands: an urgent need for improvement. BMC Infect Dis.

[6] Fortenberry JD, Brizendine EJ, Katz BP, Orr DP (2002). The role of self-efficacy and relationship quality in partner notification by adolescents with sexually transmitted infections. Arch Pediatr Adolesc Med.

[7] Fortenberry JD, McFarlane M, Bleakley A, Bull S, Fishbein M, Grimley DM, Malotte CK, Stoner BP (2002). Relationships of stigma and shame to gonorrhea and HIV screening. Am J Public Health.

[8] Lichtenstein B (2003). Stigma as a barrier to treatment of sexually transmitted infection in the American Deep South: issues of race, gender and poverty. Soc Sci Med.

[9] Golden MR, Whittington WL, Handsfield HH, Hughes JP, Stamm WE, Hogben M, Clark A, Malinski C, Helmers JR, Thomas KK (2005). Effect of expedited treatment of sex partners on recurrent or persistent gonorrhea or chlamydial infection. N Engl J Med.

[10] Kissinger P, Mohammed H, Richardson-Alston G, Leichliter JS, Taylor SN, Martin DH, Farley TA (2005). Patient-delivered partner treatment for male urethritis: a randomized, controlled trial. Clin Infect Dis.

[11] Kissinger P, Schmidt N, Mohammed H, Leichliter JS, Gift TL, Meadors B, Sanders C, Farley TA (2006). Patient-delivered partner treatment for Trichomonas vaginalis infection: a randomized controlled trial. Sex Transm Dis.

[12] Schillinger JA, Kissinger P, Calvet H, Whittington WL, Ransom RL, Sternberg MR, Berman SM, Kent CK, Martin DH, Oh MK (2003). Patient-delivered partner treatment with azithromycin to prevent repeated Chlamydia trachomatis infection among women: a randomized, controlled trial. Sex Transm Dis.

[13] Golden MR, Kerani RP, Stenger M, Hughes JP, Aubin M, Malinski C, Holmes KK (2015). Uptake and population-level impact of expedited partner therapy (EPT) on Chlamydia trachomatis and Neisseria gonorrhoeae: the Washington State community-level randomized trial of EPT. PLoS Med.

[14] Gift TL, Kissinger P, Mohammed H, Leichliter JS, Hogben M, Golden MR (2011). The cost and cost-effectiveness of expedited partner therapy compared with standard partner referral for the treatment of chlamydia or gonorrhea. Sex Transm Dis.

[15] Estcourt C, Sutcliffe L, Cassell J, Mercer CH, Copas A, James L, Low N, Horner P, Clarke M, Symonds M (2012). Can we improve partner notification rates through expedited partner therapy in the UK? Findings from an exploratory trial of Accelerated Partner Therapy (APT). Sex Transm Infect.

[16] Mohammed H, Leichliter JS, Schmidt N, Farley TA, Kissinger P (2010). Does patient-delivered partner treatment improve disclosure for treatable sexually transmitted diseases?. AIDS Patient Care STDs.

[17] Young T, de Kock A, Jones H, Altini L, Ferguson T, van de Wijgert J (2007). A comparison of two methods of partner notification for sexually transmitted infections in South Africa: patient-delivered partner medication and patient-based partner referral. Int J STD AIDS.

[18] Estcourt CS, Sutcliffe LJ, Copas A, Mercer CH, Roberts TE, Jackson LJ, Symonds M, Tickle L, Muniina P, Rait G (2015). Developing and testing accelerated partner therapy for partner notification for people with genital Chlamydia trachomatis diagnosed in primary care: a pilot randomised controlled trial. Sex Transm Infect.

[19] Centers for Disease Control and Prevention (2006). Expedited partner therapy in the management of sexually transmitted diseases.

[20] Burstein GR, Eliscu A, Ford K, Hogben M, Chaffee T, Straub D, Shafii T, Huppert J (2009). Expedited partner therapy for adolescents diagnosed with chlamydia or gonorrhea: a position paper of the Society for Adolescent Medicine. J Adolesc Health.

[21] Stekler J, Bachmann L, Brotman RM, Erbelding EJ, Lloyd LV, Rietmeijer CA, Handsfield HH, Holmes KK, Golden MR (2005). Concurrent sexually transmitted infections (STIs) in sex partners of patients with selected STIs: implications for patient-delivered partner therapy. Clin Infect Dis.

[22] Barbee LA, Soge O, Dombrowski JC, Katz D, Holmes KK, Golden MR (2015). Azithromycin-resistant Neisseria gonorrhoeae in men who have sex with men (MSM) in Seattle, Washington: 2014–2015.

[23] Golden MR, Barbee LA, Kerani R, Dombrowski JC (2014). Potential deleterious effects of promoting the use of ceftriaxone in the treatment of Neisseria gonorrhoeae. Sex Transm Dis.

[24] Kirkcaldy RD, Harvey A, Papp JR, Del Rio C, Soge OO, Holmes KK, Hook EW, Kubin G, Riedel S, Zenilman J (2014). Neisseria gonorrhoeae antimicrobial susceptibility surveillance – The Gonococcal Isolate Surveillance Project, 27 Sites, United States. MMWR Surveill Summ 2016.

[25] Kidd S, Moore PC, Kirkcaldy RD, Philip SS, Wiesenfeld HC, Papp JR, Kerndt PR, Venkatasubramanian L, Ghanem KG, Hook EW (2015). Comparison of antimicrobial susceptibility of urogenital Neisseria gonorrhoeae isolates obtained from women and men. Sex Transm Dis.

[26] Kerani RP, Fleming M, DeYoung B, Golden MR (2011). A randomized, controlled trial of inSPOT and patient-delivered partner therapy for gonorrhea and chlamydial infection among men who have sex with men. Sex Transm Dis.

[27] Stephens SC, Bernstein KT, Katz MH, Philip SS, Klausner JD (2010). The effectiveness of patient-delivered partner therapy and chlamydial and gonococcal reinfection in San Francisco. Sex Transm Dis.

[28] Bauer HM, Wohlfeiler D, Klausner JD, Guerry S, Gunn RA, Bolan G, California STDCA (2008). California guidelines for expedited partner therapy for Chlamydia trachomatis and Neisseria gonorrhoeae. Sex Transm Dis.

[29] Jones HE, Lippman SA, Pinho AA, Luppi CG, van de Wijgert JH, Diaz J (2006). Partner-delivered medication for sexually transmitted infections: findings from Brazil. Gac Med Mex..

[30] Alam N, Chamot E, Vermund SH, Streatfield K, Kristensen S (2010). Partner notification for sexually transmitted infections in developing countries: a systematic review. BMC Public Health..

[31] Faxelid EA, Ramstedt KM (1997). Partner notification in context: Swedish and Zambian experiences. Soc Sci Med.

[32] Hogben M, Hood J (2011). Acquired skills in sexually transmitted disease prevention: partner services and tailoring interventions to populations. Sex Transm Dis.

[33] Clark JL, Long CM, Giron JM, Cuadros JA, Caceres CF, Coates TJ, Klausner JD (2007). Partner notification for sexually transmitted diseases in Peru: knowledge, attitudes, and practices in a high-risk community. Sex Transm Dis.

[34] Clark JL, Perez-Brumer A, Salazar X (2015). “Manejar la Situacion”: partner notification, partner management, and conceptual frameworks for HIV/STI control among MSM in Peru. AIDS Behav..

[35] Low N, Broutet N, Adu-Sarkodie Y, Barton P, Hossain M, Hawkes S (2006). Global control of sexually transmitted infections. Lancet.

[36] Blair CS, Segura ER, Perez-Brumer AG, Sanchez J, Lama JR, Clark JL (2016). Sexual orientation, gender identity and perceived source of infection among men who have sex with men (MSM) and transgender women (TW) recently diagnosed with HIV and/or STI in Lima, Peru. AIDS Behav..

[37] Cambou MC, Perez-Brumer AG, Segura ER, Salvatierra HJ, Lama JR, Sanchez J, Clark JL (2014). The risk of stable partnerships: associations between partnership characteristics and unprotected anal intercourse among men who have sex with men and transgender women recently diagnosed with HIV and/or STI in Lima, Peru. PLoS One.

[38] Perez-Brumer AG, Oldenburg CE, Segura ER, Sanchez J, Lama JR, Clark JL. Anonymous partnerships among MSM and transgender women (TW) recently diagnosed with HIV and other STIs in Lima, Peru: an individual-level and dyad-level analysis. Sex Transm Infect. 2016;Feb 24. doi: 10.1136/sextrans-2015-052310. 10.1136/sextrans-2015-052310 PMC4996769 26912910

[39] Fleming E, Hogben M (2017). Assessing different partner notification methods for assuring partner treatment for gonorrhea: looking for the best mix of options. J Public Health Manag Pract.

[40] Ferreira A, Young T, Mathews C, Zunza M, Low N (2013). Strategies for partner notification for sexually transmitted infections, including HIV. Cochrane Database Syst Rev..

[41] Silva-Santisteban A, Raymond HF, Salazar X, Villayzan J, Leon S, McFarland W, Caceres CF (2012). Understanding the HIV/AIDS epidemic in transgender women of Lima, Peru: results from a sero-epidemiologic study using respondent driven sampling. AIDS Behav.

[42] Sanchez J, Lama JR, Kusunoki L, Manrique H, Goicochea P, Lucchetti A, Rouillon M, Pun M, Suarez L, Montano S (2007). HIV-1, sexually transmitted infections, and sexual behavior trends among men who have sex with men in Lima, Peru. J Acquir Immune Defic Syndr..

[43] Castillo R, Konda KA, Leon SR, Silva-Santisteban A, Salazar X, Klausner JD, Coates TJ, Caceres CF (2015). HIV and sexually transmitted infection incidence and associated risk factors among high-risk MSM and male-to-female transgender women in Lima, Peru. J Acquir Immune Defic Syndr..

[44] Lama JR, Lucchetti A, Suarez L, Laguna-Torres VA, Guanira JV, Pun M, Montano SM, Celum CL, Carr JK, Sanchez J (2006). Association of herpes simplex virus type 2 infection and syphilis with human immunodeficiency virus infection among men who have sex with men in Peru. J Infect Dis.

[45] Clark J, Salvatierra J, Segura E, Salazar X, Konda K, Perez-Brumer A, Hall E, Klausner J, Caceres C, Coates T (2013). Moderno love: sexual role-based identities and HIV/STI prevention among men who have sex with men in Lima, Peru. AIDS and Behavior..

[46] Clark JL, Perez-Brumer AG, Segura ER, Salvatierra HJ, Sanchez J, Lama JR (2016). Anticipated notification of sexual partners following STD diagnosis among men who have sex with men and transgender women in Lima, Peru: a mixed methods analysis. PLoS One.

[47] Gardner EM, McLees MP, Steiner JF, Del Rio C, Burman WJ (2011). The spectrum of engagement in HIV care and its relevance to test-and-treat strategies for prevention of HIV infection. Clin Infect Dis.

[48] Mugavero MJ, Amico KR, Horn T, Thompson MA (2013). The state of engagement in HIV care in the United States: from cascade to continuum to control. Clin Infect Dis.

[49] Alsdurf H, Hill PC, Matteelli A, Getahun H, Menzies D (2016). The cascade of care in diagnosis and treatment of latent tuberculosis infection: a systematic review and meta-analysis. Lancet Infect Dis.

[50] Parsons JT, Rendina HJ, Lassiter JM, Whitfield TH, Starks TJ, Grov C (2017). Uptake of HIV pre-exposure prophylaxis (PrEP) in a national cohort of gay and bisexual men in the United States: the Motivational PrEP Cascade. J Acquir Immune Defic Syndr.

[51] Tang EC, Segura ER, Clark JL, Sanchez J, Lama JR (2015). The syphilis care cascade: tracking the course of care after screening positive among men and transgender women who have sex with men in Lima, Peru. BMJ Open..

[52] Lichtenstein B, Schwebke JR (2005). Partner notification methods for African American men being treated for trichomoniasis: a consideration of Main Men, Second Hitters, and Third Players. Med Anthropol Q.

[53] Singer MC, Erickson PI, Badiane L, Diaz R, Ortiz D, Abraham T, Nicolaysen AM (2006). Syndemics, sex and the city: understanding sexually transmitted diseases in social and cultural context. Soc Sci Med.

[54] Katz DA, Dombrowski JC, Kerani RP, Aubin MR, Kern DA, Heal DD, Bell TR, Golden MR (2016). Integrating HIV testing as an outcome of STD partner services for men who have sex with men. AIDS Patient Care STDs.

